# *PSMB7* is associated with anthracycline resistance and is a prognostic biomarker in breast cancer

**DOI:** 10.1038/sj.bjc.6605478

**Published:** 2009-12-15

**Authors:** G Munkácsy, R Abdul-Ghani, Z Mihály, B Tegze, O Tchernitsa, P Surowiak, R Schäfer, B Györffy

**Affiliations:** 1 Joint Research Laboratory of the Hungarian Academy of Sciences and the Semmelweis University, Semmelweis University 1st Department of Pediatrics, Budapest, Hungary; 2 Biochemistry Department, Faculty of Medicine, Al-Quds University, East Jerusalem, Palestine; 3 Laboratory of Molecular Tumor Pathology, Institute of Pathology, Charité Universitätsmedizin Berlin, Berlin, Germany; 4 Chair and Department of Histology and Embryology, University School of Medicine, Wrocław, Poland

**Keywords:** *PSMB7*, breast cancer, biomarker, doxorubicin, RNA interference, gene expression

## Abstract

**Background:**

To date individual markers have failed to correctly predict resistance against anticancer agents in breast cancer. We used gene expression patterns attributable to chemotherapy-resistant cells to detect potential new biomarkers related to anthracycline resistance. One of the genes, *PSMB7*, was selected for further functional studies and clinical validation.

**Methods:**

We contrasted the expression profiles of four pairs of different human tumour cell lines and of their counterparts resistant to doxorubicin. Observed overexpression of *PSMB7* in resistant cell lines was validated by immunohistochemistry. To examine its function in chemoresistance, we silenced the gene by RNA interference (RNAi) in doxorubicin-resistant MCF-7 breast cancer cells, then cell vitality was measured after doxorubicin treatment. Microarray gene expression from GEO raw microarray samples with available progression-free survival data was downloaded, and expression of *PSMB7* was used for grouping samples.

**Results:**

After doxorubicin treatment, 79.8±13.3% of resistant cells survived. Silencing of *PSMB7* in resistant cells decreased survival to 31.8±6.4% (*P*>0.001). A similar effect was observed after paclitaxel treatment. In 1592 microarray samples, the patients with high *PSMB7* expression had a significantly shorter survival than the patients with low expression (*P*<0.001).

**Conclusion:**

Our findings suggest that high *PSMB7* expression is an unfavourable prognostic marker in breast cancer.

The major cause of cancer therapy failure is either primary drug resistance or the development of secondary resistance against currently available antineoplastic agents. Many different mechanisms of drug resistance have been identified, including the overexpression of members of the family of adenosine triphosphate-binding cassette (ABC)-transporters such as P-glycoprotein (*ABCB1*), the multidrug resistance-associated proteins MRP1 and MRP2 and the mitoxantrone resistance protein/breast cancer resistance protein (Gottesman *et al*, 2002). Drug resistance is also mediated by defects in intrinsic cellular safeguard mechanisms capable of activating apoptosis. The loss of programmed cell death induced by antitumour drugs can be achieved through disruption of regulators of DNA damage signalling, such as *p53* and *bcl-2*. Moreover, alterations in cell cycle checkpoints, repair of damaged cellular targets and various additional more or less well-characterised mechanisms can contribute to the resistance.

The anthracycline antibiotic doxorubicin (adriamycin) is widely used for treatment of breast, ovarian, bronchogenic and gastric solid tumours, lymphomas and leukaemias (Martin *et al*, 2003). Triple negative (ER-, PR-, HER2-) breast patients receive anthracycline-based chemotherapeutic agents in monotherapy or in combination protocols. Although a number of different mechanisms have been proposed for the cytotoxic effect of anthracyclines, the primary mechanism of drug action is likely to be the inhibition of DNA biosynthesis through topoisomerase II binding and consequently conferring an S/G2 cell cycle arrest (Gewirtz, 1999). As a topoisomerase inhibitor, it potentially induces apoptosis in normal cells as well (Sabourin and Osheroff, 2000).

In several reports, authors applied DNA array technologies to find genes correlated with doxorubicin resistance (Ayers *et al*, 2004; Kang *et al*, 2004; Folgueira *et al*, 2005; Gianni *et al*, 2005; Cleator *et al*, 2006; Gyorffy *et al*, 2006; Hess *et al*, 2006). In our earlier study, we used gene expression patterns of closely related chemotherapy-resistant and -sensitive parental cancer cell lines to identify discriminatory genes associated with drug resistance. The use of a set of different cell lines for the identification of discriminating genes allows a tissue-independent application for predicting therapy response. We applied our model to predict sensitivity in a set of 44 breast cancer samples. The patient group characterised by the gene expression profile similar to that of drug-sensitive cell lines exhibited over 50% longer survival than the group exhibiting the profile characteristic of resistant cells (Gyorffy *et al*, 2005). Overall, in these gene expression signatures, a considerable number of genes were associated with doxorubicin resistance. However, it is desirable to discriminate markers associated with treatment response and markers responsible for resistance.

The proteasome is a multicatalytic proteinase complex with a highly ordered ring-shaped 20S core structure. The core structure is composed of 4 rings of 28 non-identical subunits; 2 rings are composed of 7 *α* subunits and 2 rings are composed of 7 *β* subunits. Proteasomes are distributed throughout eukaryotic cells at a high concentration and cleave damaged and needless peptides in an ATP/ubiquitin-dependent process in a non-lysosomal pathway. Proteasome inhibitors are drugs blocking the action of cellular complexes that break down proteins. They have an antitumour activity in cell culture, and induce apoptosis by disturbing regulation of cell cycle proteins (Adams *et al*, 1999). Bortezomib was the first proteasome inhibitor, which is already used in clinics as a chemotherapy agent (Kane *et al*, 2006). Bortezomib effectively eradicates myelome multiplex by activating the stress apoptosis signal of endoplasmic reticulum (Schewe and guirre-Ghiso, 2009). So far, several studies examined its effect on proteasomes in different circumstances (Ludwig *et al*, 2005; Montagut *et al*, 2005; Montagut *et al*, 2006; Sterz *et al*, 2008). Proteasome inhibitors might also increase chemosensitivity. Cotreatment of doxorubicin and bortezomib can conversely increase the efficiency of each other (Ciolli *et al*, 2008). However, to date the direct association with doxorubicin resistance as well as the potential prognostic or predictive value of selected proteasome subunits has not been investigated.

In this study, our intention was to identify gene expression signatures obtained from resistant cells in long-term culture, untreated sensitive parental cells and parental cells after short-term drug administration. On the basis of relevance in clinical samples, we selected one proteasome subunit, the proteasome unit *β*-type 7 (PSMB7) for further examination using RNA interference (RNAi) to validate its function in doxorubicin resistance. Our aim was also to identify effects of paclitaxel cross-resistance on the resistant cell line. Finally, microarray data from 1592 patients were used to examine the clinical relevance of PSMB7.

## Materials and methods

### Cell lines

The doxorubicin/daunorubicin-resistant derivatives of human gastric carcinoma cell line EPG85-257P, pancreatic carcinoma cell line EPP85-181P, colon carcinoma cell line HT-29 and breast cancer cell line MCF-7 were established in our laboratory as described earlier (Lage and Dietel, 2002). Cells were cultured in Leibovitz L-15 medium (Sigma-Aldrich, Budapest, Hungary) supplemented with 10% foetal calf serum (Life Sciences, Grand Island, NY, USA), 1 mM L-glutamine, 80 IE/l insulin, 2.5 mg l^–1^ transferrin, 1 g l^–1^ glucose, 1.1 g l^–1^ NaHCO_3_, 1% minimal essential vitamins and 20 000 kIE l^–1^ trasylol in a humified atmosphere containing 5% CO_2_ at 37°C. Culture media of resistant cell lines were supplemented with daunorubicin for HT-29 (0.125 *μ*g ml^–1^), EPP85-181 (2.5 *μ*g ml^–1^) and EPG-257 (2.5 *μ*g ml^–1^) and doxorubicin (0.05 *μ*g ml^–1^) for MCF-7 cells (Farmitalia Carlo Erba, Freiburg, Germany). The 24 h treatment of drug-sensitive cells was performed at the identical drug concentrations.

### Sensitivity of cells to doxorubicin and paclitaxel

Cell proliferation assay (Cell Proliferation Kit I (MTT) Roche, IN, USA,) was used to monitor sensitivity of doxorubicin-resistant cell lines. Protocol was carried out as directed by the manufacturer. Briefly, 5000 cells per well in a microplate were plated, and drug was added after an overnight incubation in increasing grade of 0.0001x–100x of the clinically administered dose (doxorubicin: 0.02 *μ*g ml^–1^, paclitaxel: 0.025 *μ*g ml^–1^) in triplicates. Six days later, MTT (3-[4, 5-dimethylthiazolyl-2]-2, 5-diphenyltetrazolium bromide) was added to each well for 4 h. After this incubation period, the formed purple formazan salt crystals were solubilised and quantified 1 day later with the use a BioTek PowerWave XS Microplate Spectrophotometer.

### Autofluorescence of doxorubicin

A total of 50 000 sensitive MCF-7 cells per well were plated in triplicate in a 96-well plate for four different conditions: (1) cells treated by doxorubicin, (2) untreated cells, (3) doxorubicin in L15 medium without cells and (4) L15 medium alone. Doxorubicin treatment (0.2 *μ*g ml^–1^) was carried out on the second day for 1.5 h. MTT staining and reading the results were performed as described above. Differences were assessed by using a *t*-test.

### RNA isolation and cDNA synthesis

RNA was isolated from 1 × 10^7^ cells in logarithmic growth phase using the Qiagen RNeasy Mini Kit following the manufacturer's protocol (Qiagen GmbH, Hilden, Germany). Isolated total RNA was quantified by UV-spectroscopy and quality checked by analysis on a LabChip (BioAnalyzer, Agilent Technologies, Santa Clara, CA, USA). RNA samples were stored at −80°C. cDNA was synthesised from 5 *μ*g total RNA by firstly annealing to 5 pmol *μ*l^–1^ HPLC purified T7- (dT)24 primer (MWG-Biotech, Ebersberg, Germany) at 70°C for 10 min. Second, reverse transcription, second-strand synthesis and cleanup of double-stranded cDNA was performed according to the protocols provided by Affymetrix (http://www.affymetrix.com/index.affx). Synthesis of biotin-labelled cRNA was performed using the BioArray High Yield RNA Transcription kit (Enzo Diagnostics, Farmingdale, NY, USA). cRNA concentration was determined by UV-spectroscopy and the distribution of cRNA fragment size was checked on a LabChip (BioAnalyzer, Agilent Technologies).

### Hybridization protocol

The fragmented cRNA was hybridised to the HG-U133A arrays (Affymetrix, Santa Clara, CA, USA) in a hybridization oven at 45°C for 16 h. Subsequent washing and staining of arrays was performed using the GeneChip fluidics station protocol EukGE-WS2. Finally, probe arrays were scanned using the GeneChip System confocal scanner (Hewlett-Packard, Santa Clara, CA, USA). For each cell line triplicates (sensitive, resistance and treated), hybridization was made once, so altogether 12 hybridizations were made. The microarray data can be downloaded from GEO (accession no. GSE3926).

### Pre-processing of microarray data

Quality control analysis was performed according to the suggestions of The Tumor Analysis Best Practices Working Group (Hoffman *et al*, 2004). Scanned images were inspected for artefacts. All RNA targets included in the analysis exhibited present calls of >25% and were not degraded. We have applied RMA (Irizarry *et al*, 2003) for the normalization of hybridization intensities. The pre-processing was performed using Bioconductor packages in the R statistical environment. RMA is one of the most common array normalization methods supplying cross-project normalization with good specificity and excellent sensitivity.

### Feature selection

**Figure 1:**
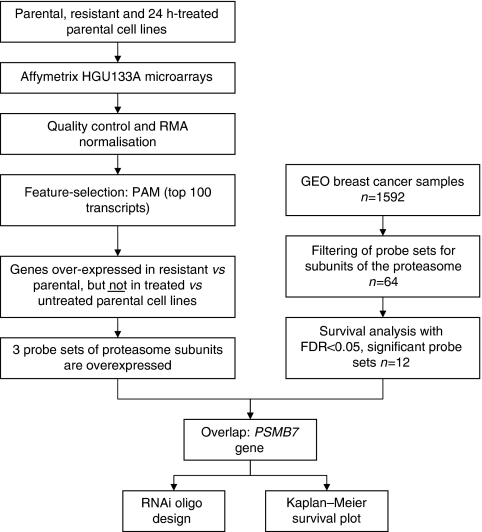
Overview of the statistical analysis.

We arranged the complete data set into three classes according to the resistance and treatment properties of the cell lines. We compared drug-sensitive parental, doxorubicin/daunorubicin-treated parental and resistant cell lines. For identifying discriminating genes, the Prediction Analysis for Mircorarrays (PAM v.1.23) package was used as described earlier (Tibshirani *et al*, 2002). PAM uses soft thresholding for producing a shrunken centroid, which allows the selection of genes with high discriminative potential. An overview of the applied statistical approach is presented in Figure 1. We decided to pick the top 100 genes from each comparison for the selection of the best discriminatory group of genes. Then genes were identified, which discriminate between parental and treated parental cell lines (reflecting effect of the drug treatment) and resistant and parental cell lines (reflecting genes of resistance).

### Additional analyses

Hierarchical clustering was performed using the Genesis software (Sturn *et al*, 2002). Before clustering, we performed a second normalization at the gene level to set the average expression of each transcript to 0 to present gene inductions or repressions of identical magnitude as numerically equal. Gene annotations were performed using the Netaffx Analysis Centre (http://www.affymetrix.com/analysis/index.affx).

### Immunohistochemistry

Cells were grown on microscopic slides and fixed in ice-cold methanol–acetone mixture (1 : 1). Activity of endogenous peroxidase was blocked by incubation in 1% H_2_O_2_. Immunohistochemical stainings were carried out in triplicate with primary antibody polyclonal antiserum against *PSMB7* (dilution 1 : 200; GenWay Biotech, Inc., CA, USA). Subsequently, slides were incubated with secondary biotinylated antibodies anti-mouse, rabbit and goat; optimally prediluted from LSAB+, HRP Kit (DakoCytomation, Glostrup, Denmark); followed by optimally prediluted streptavidin–biotinylated peroxidase complex (LSAB+, HRP, DakoCytomation) and the chromogen NovaRed (Vector Laboratories, Peterborough, UK) at room temperature. The intensity of antigen expression was measured using the digital imaging system Lucia-G/F (Nikon, Tokyo, Japan). Mean saturation feature was determined in four microscopic fields at 200-fold magnification. Nucleus staining was performed by DAPI (4′,6-diamidino-2-phenylindole).

### Oligos for RNAi

**Figure 2 fig2:**
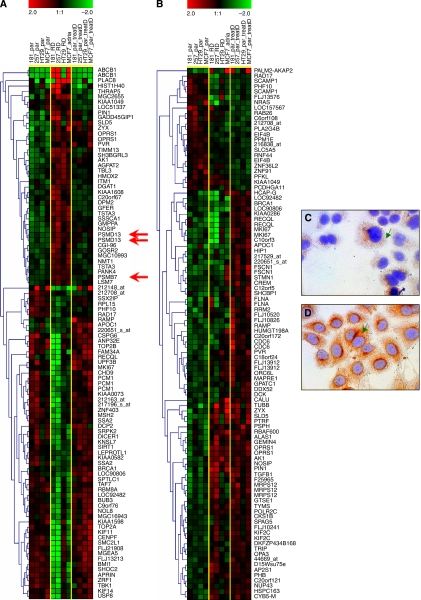
Discriminating transcripts: (**A**) hierarchical clustering of all three groups (parental, treated parental, resistance cell line) against each other and (**B**) hierarchical clustering of parental *vs* treated parental cell lines. Red arrows show appearance of three probe sets measuring proteasome subunits on resistance-associated gene list. Immunohistochemical localization of *PSMB7* expression in sensitive (**C**) and resistant cells (**D**). Reactions of cytoplasmic localization were obtained for *PSMB7* (green arrows).

**Figure 3:**
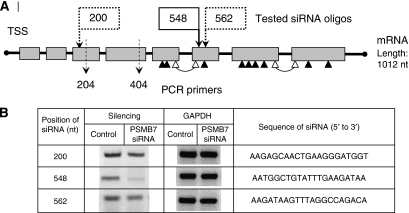
(**A**) Schematic view of *PSMB7* gene with its eight exons. Splitting positions of tested siRNA oligos are shown (↓); the most effective siRNA is marked by continuously bordered box. Binding location of designed PCR primers specific for *PSMB7* is shown under the gene by a broken line (↓). Positions of probe sequences of probeset 200786_at on Affymetrix HG-U133A array are also shown (▴, match probes; ▵, junction probes, overlapping two neighbouring exons). (**B**) Details of designed siRNA oligos. One out of three siRNA oligos (degrading at 548 nucleotide (nt)) showed effective silencing compared with siRNA-untreated control. *GAPDH* was used as internal control for both untreated control and siRNA-treated samples. The effect of silencing was 87% for siRNA binding at position 548.

Target of siRNA oligos for *PSMB7* were designed using siRNA Target Finder (http://www.ambion.com) and siDESIGN Center (http://www.dharmacon.com) softwares. DNA oligonucleotide templates for three target mRNAs were ordered (Csertex Kft, Budapest, Hungary), then were synthesised by Silencer siRNA Construction Kit (Applied Biosystems, Darmstadt, Germany) using the manufacturer's instruction. Synthesised siRNA duplex concentrations were measured by NanoDrop ND-1000 spectrophotometer (BCM, Houston, TX, USA) then diluted as needed. PCR primers were designed by Primer3 software (http://frodo.wi.mit.edu/) and their binding site was verified by NCBI BLAST (http://blast.ncbi.nlm.nih.gov/Blast.cgi). Out of three synthesised siRNA oligos, one silenced effectively *PSMB7* better than 80%. Figure 3 shows binding location of every synthesised oligos and PCR primers (effective siRNA is framed by continuous line). Effectiveness of siRNA oligos was validated by RT–PCR [Fig fig2](Figure 3B).

### SiRNA transfection of MCF-7 cell lines

Transfection was carried out with 5 *μ*l SiPORT *NeoFX* Transfection Reagent (Applied Biosystems, Budapest, Hungary) in six-well plates. A total of 230 000 cells were plated into each well. The final concentration of siRNA was 10 nM in 2.5 ml serum and antibiotic-free medium. After 24 h of incubation, medium was washed twice with sterile PBS, then normal growth medium was added. Total RNA was extracted in the 48th h after transfection with Qiagen RNeasy Mini Kit as described earlier, cDNA synthesis and DNA amplification were carried out by OneStep RT–PCR Kit (Qiagen GmbH) with gene-specific PCR primers. *GAPDH* was used as internal control. We used 24 amplification cycles for amplification. Amplification products were separated on 2% agarose gel stained with ethidium-bromide. The effect of silencing was calculated by Adobe Photoshop CS2 software.

### Combination of RNAi and chemotherapy treatment

To investigate the function of selected gene in chemoresistance, we combined RNAi and drug treatment. *PSMB7* overexpressed in resistant cells was silenced in doxorubicin-resistant MCF-7 cell lines (MCF-7-RAdr); 24 h after transfection, the reaction was stopped by replacing the medium with normal growth medium containing drug in concentration of 0.2 *μ*g ml^–1^. Cells were trypsinised on the 72nd h and counted by CASY DT Cell Counter (Innovatis AG, Reutlingen, Germany). We used negative siRNA-treated (AllStars Negative Control siRNA, Qiagen GmbH) MCF-7-RAdr cells, siRNA-untreated MCF-7-RAdr cells and siRNA-untreated MCF-7 cells for controls with their doxorubicin-treated analogue. For a statistical analysis, the number of living cells was counted in each well. Experiments were carried out three times, and each well was measured three times in each experiment. The number of drug-treated cells was normalised to untreated cells in siRNA-treated, negative siRNA-treated and siRNA-untreated wells. The *t*-test was used for analysis of difference between groups. Significance level was set at *P*=0.05.

### Validation on clinical samples

For in silico validation of the genes, a database containing processed GEO microarray samples was established as described earlier (Gyorffy and Schafer, 2009). Dataset includes GSE12276, GSE16391, GSE12093, GSE11121, GSE9195, GSE7390, GSE6532, GSE5327, GSE4922, GSE3494, GSE2990, GSE2034 and GSE1456. First, genes were filtered to include only subunits of the proteasome (*n*=64). Then a survival analysis for each of these probe sets were performed using BRB Arraytools 3.8.0-*β*_1 package (developed by Dr Richard Simon and Amy Peng Lam, available at http://linus.nci.nih.gov/BRB-ArrayTools.html). The false discovery rate (FDR) was set below 0.05. Finally, Kaplan–Meier plots were drawn to illustrate the effect on survival in these patients.

## Results

### Identification of differentially regulated genes

To identify genes associated with the resistance to doxorubicin/daunorubicin, we contrasted gene expression profiles of closely related drug-resistant and -sensitive cell lines derived from breast, pancreatic, colon and gastric cancer. For microarray analysis, RNA was prepared from non-treated parental cells, from parental cells treated with doxorubicin for 24 h and from resistant derivatives cultured continuously in the presence of doxorubicin at a concentration that completely eliminated the sensitive cells. The complete data set comprising raw data and Affymetrix .CEL files is available in the GEO database (http://www.ncbi.nlm.nih.gov/geo/) using the GEO accession number GSE3926. The prediction analysis of microarrays was performed to pick the top 100 genes differentially expressed in each sample set independent of tissue origin. To visualise discriminatory expression changes, we clustered the top genes (see Figure 2A and B). In these, three probe sets representing proteasome subunits were expressed stronger in doxorubicin-resistant cell lines, but not in doxorubicin-treated parental cells (see arrows in Figure 2).

### Autofluorescence of doxorubicin

Examining the effect of doxorubicin autofluorescence on MTT results, there was no difference between doxorubicin-treated and -untreated cells (*P*=0.38). Fluorescence of doxorubicin supplemented L15 medium differed significantly from doxorubicin-treated cells (*P*<0.01), but did not differ from untreated L15 medium (*P*=0.065).

### Prognostic potential of 64 proteasome subunits

The normalised gene expression of the GEO-downloaded 1592 samples was filtered to include only the proteasome subunits present on the HG-U133A microarrays. The prognostic potential for all 64 probe sets was computed by dividing the patients into over- and underexpressed group as compared with the median (thus, 531 patients were in each group). The analysis resulted in 12 significant genes below an FDR of 0.05. Of these, only one was also detected by the microarray analysis (*PSMB7*), which was further investigated. Immunocytochemistry confirmed the overexpression of *PSMB7* as observed on the microarrays (Figure 2C and D).

### Survival of cells after gene silencing and treatment with doxorubicin and paclitaxel

To examine the function of the *PSMB7* gene in doxorubicin resistance, we combined gene silencing with drug treatment to assess its effect on cell survival. Viability of cells without doxorubicin treatment was better in both siRNA-untreated and siRNA-treated wells compared with cells with doxorubicin treatment. A specific cytotoxic effect of negative control siRNA was negligible as compared with the effect of gene-specific siRNA.

**Figure 4:**
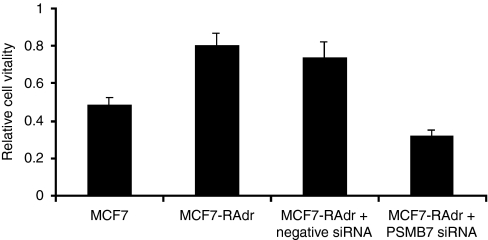
The effect of silencing of *PSMB7* on doxorubicin resistance in MCF-7 cells.

Data after normalization are presented in Figure 4; 79.8±13.3% of resistance cells survived after doxorubicin treatment. Combined with gene silencing, only 31.8±6.4% of the MCF-7-RAdr cells survived. The significance between siRNA-treated and siRNA-untreated MCF-7-RAdr cells after doxorubicin treatment was *P*>0.001. After doxorubicin treatment, 48.3±8.1% of sensitive cells survived. A total of 73.3% of negative control siRNA-treated cells survived. After combination of paclitaxel treatment and gene silencing, 22.6±4% of the MCF-7-RAdr cells survived compared with siRNA-untreated cells, whereas 43.8±6% of sensitive cells survived (*data not shown*). Relative cell vitality of siRNA-treated and siRNA-untreated MCF-7-RAdr cells after paclitaxel treatment differed significantly (*P*=0.03).

### Biomarker potential of *PSMB7* in clinical samples

**Figure 5:**
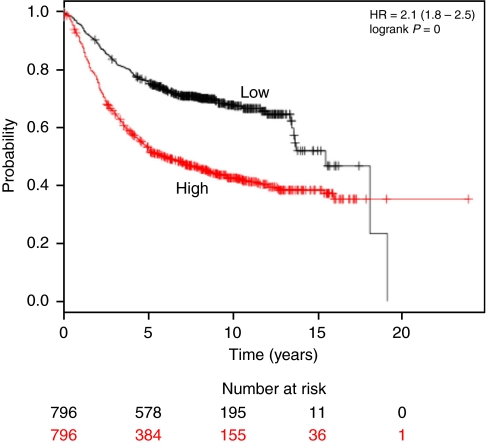
Kaplan–Meier of survival of the 1592 breast cancer patients grouped by the expression of *PSMB7* (200786_at probe set) above or below the median.

To assess the prognostic power of *PSMB7* gene in resistance, clinical samples were used to validate the results of cell culture model. We grouped the 1592 breast cancer patients on the basis of the expression of *PSMB7* using the Affymetrix HGU133A probe set 200786_at. Of these, 963 out of 1220 patients were ER positive and 187 out of 1156 lymph node positive. The average relapse-free survival was 6.42 years. Unfortunately, detailed treatment history was generally not available. Patients above the median had a significantly shorter survival than patients below the median (*P*<0.001) (Figure 5).

## Discussion

In our study we examined the function of the proteasome subunit *PSMB7* gene in drug resistance in breast cancer cell line and in breast cancer patients. So far, no earlier study has risen up concerning drug resistance neither for *PSMB7* gene nor any of proteasome subunits. In a cell culture model with a combination of RNAi and drug treatment, we validated the causative function of this gene in chemoresistance. The examination of 1592 breast cancer patient showed that overexpression of this gene is associated with poor prognostic outcome.

At the preliminary phase of this study, we contrasted the expression profiles of four pairs of different human tumour cell lines of gastric, pancreatic, colon and breast origin, their counterparts resistant to the topoisomerase inhibitors daunorubicin or doxorubicin and the sensitive parental cells after a 24 h-chemotherapy treatment using Affymetrix HG-U133A microarrays. We also performed additional analyses to exclude the influence of doxorubicin autofluorescence (Karukstis *et al*, 1998) on our results. Finally, we identified the top transcripts associated with doxorubicin/daunorubicin resistance, but not with treatment response. So far, the majority of microarray-based chemoresistance-associated gene sets concentrated on finding genes associated with resistance rather than on the difference between drug response and drug resistance. Surprisingly, we have found genes such as proteasome subunits coding *PSMB7* and *PSMD13* having function in the mechanism of resistance that earlier have not been described. Of these, only the expression of *PSMB7* was able to predict survival in clinical samples and was, therefore, further investigated using RNAi.

The *Homo sapiens* gene *PSMB7* encodes proteasome (also called prosome or macropain) subunit *β*-type, 7. In the Haloarchaeon Haloferax Volcanii proteasomal 20S, components are required for cell growth (Zhou *et al*, 2008). Proteasome and tRNA modification genes are co-transcribed, revealing that a number of additional enzymes are co-regulated with proteasomes at the transcriptional level in the same species (Gil *et al*, 2007). Other animal experiments related to proteasome and immunology were made on Japanese pufferfish (Clark *et al*, 2001) and zebrafish (Michalova *et al*, 2000). The importance of monitoring proteasomes has emerged in the last couple of years as it has an important function in the degradation of many proteins involved in cell cycle regulation, apoptosis and angiogenesis. As these pathways are fundamental for cell survival and proliferation, particularly in cancer cells, the inhibition of proteasome might deliver an attractive potential anticancer therapy. Proteolysis is conducted by 20S proteasomes, complexes of 28 subunits arranged as a cylinder in four heteroheptameric rings: *α*-1 to -7, *β*-1 to -7, *β*-1 to -7 and *α*-1 to -7. The catalytic subunits are *β*-1 (*PSMB6*), *β*-2 (*PSMB7*) and *β*-5 (*PSMB5*). Three additional subunits, *β*-1i (*PSMB9*), *β*-2i (*PSMB10*) and *β*-5i (*PSMB8*), are induced by *γ*-interferon (IFNG) and are preferentially incorporated into proteasomes to make immunoproteasomes. *PSMB11*, or *β*-5t, is a catalytic subunit expressed exclusively in cortical thymic epithelial cells (Murata *et al*, 2007).

The function of one of the catalytic subunits, the *PSMB7* gene in cancer is still an undiscovered spot of molecular biology. Its overexpression is described in colorectal carcinomas, in both cytoplasmic and nuclear region (Rho *et al*, 2008). After adaptation of proteasome inhibitor expression of *PSMB5* increased, but other components of proteasome such as *PSMB7* did not (Oerlemans *et al*, 2008). Looking for tissue-specific alteration in proteasome units, researchers treated mice with 3*H*-1,2-dithiole-3-thione (D3T), which functions as a cancer preventive agent proved both in animal and human studies; 24 h later, expression of the 20S catalytic core subunits *PSMB5*, *PSMB6* and *PSMB7* were increased in liver, lung, small intestine and colon of mice (Kwak *et al*, 2007a). Elevated expression of proteasome catalytic subunits led to increase in proteasomal peptidase activities in these tissues. Oral administration of D3T also exerted a pharmacodynamic action in some brain regions of these mice and proteasomal peptidase activities were significantly elevated in the cerebral cortex–hippocampus. These results indicate that increased proteasome expression by inducers may have a function in protection/attenuation of protein aggregate-mediated disorders (Kwak *et al*, 2007b).

After *in vitro* inspection, we further validated the discriminating function of differential *PSMB7* expression in 1592 publicly available microarrays . By dividing the patients as having over- or underexpressed *PSMB7* as compared with the median, we achieved high significance. However, our approach is limited because of inadequate treatment information. A future study for fine tuning of *PSMB7* expression with RT–PCR in a large clinical sample collection with detailed clinical follow-up could identify exact cut offs for thresholds, which could validate and enhance its discriminative potential in clinical use.

We might consider the idea to silence overexpressed *PSMB7* as part of a chemotherapy. However, despite promising *in vitro* results, the establishment of effective RNAi conditions *in vivo* is still complicated. A major impediment to the clinical use of RNAi therapy is the need to deliver these macromolecules to each and every cancer cell to trigger a direct and specific effect. Several studies have shown *in vivo* efficacy in the delivery of siRNAs using various strategies such as complexing siRNAs with cationic lipids, nanoparticles, polyethyleneimine, cyclodextrin, chitosan and collagen (Nguyen *et al*, 2008). Xenograft mouse model was also effectively used in several studies (Xiao *et al*, 2008; Xie *et al*, 2008; Zhang *et al*, 2008, 2009; Shi *et al*, 2009). In human beings, RNAi is already in clinical use in therapy of macula degeneration and respiratory syncytial virus infection, but so far no effective RNAi-based inhibition of tumour progression is available.

As it was examined in the last decades, overexpression of individual genes can be associated with resistance against given agents. *ABCB1* gene strongly correlates to chemoresistance (Clarke *et al* 1992; Kamata *et al*, 2008; Larbcharoensub *et al*, 2008; Shi *et al*, 2008), *TOP2A* is a potential gene for predicting anthracyclin resistance (Molina *et al*, 2005; Tanner *et al*, 2006; Harris *et al*, 2007; Mano *et al*, 2007). Expression of metallothioneins is linked to tamoxifen resistance (Surowiak *et al*, 2004), and the gene *Tau* is a predictor of resistance against neoadjuvant paclitaxel therapy (Rouzier *et al*, 2005; Andre *et al*, 2007). The favourable outcome of this study suggests that *PSMB7* gene has an important function in predicting both doxorubicin and paclitaxel resistance.

In summary, our findings support the function of the proteasome in the development of chemotherapy resistance. High *PSMB7* expression is an unfavourable prognostic marker in breast cancer.
